# Implementing an encrypted display with the electron-induced colour router array

**DOI:** 10.1038/s41377-025-01889-9

**Published:** 2025-06-16

**Authors:** Hyoseok Park, Minsu Park, Yeonsang Park

**Affiliations:** Department of Physics, Chungnam National University, Daejeon, 34134 South Korea

**Keywords:** Optical metrology, Integrated optics

## Abstract

Electron-induced colour routers actively manipulate dichromatic photon momentum at deep subwavelength scales, enabling programmable encrypted displays with enhanced security and high integration for advanced photonic applications.

Metasurfaces serve as innovative platforms for precise photon propagation modulation, enabling control over light’s characteristics in both frequency and spatial domains. For example, metasurfaces, composed of nanometer-scale patterns, can manipulate the phase, amplitude, and polarization of light to steer its propagation in desired directions^1–5^. Nanoantennas enhance localized electromagnetic fields at specific wavelengths, modulating the propagation direction of photons or amplifying specific optical phenomena. Particularly, plasmonic nanoantennas leverage the interaction between electromagnetic waves and metal surfaces to achieve high-resolution optical signal control^6–9^. These technologies find applications in high-speed data transmission for optical communications, photon encoding for quantum information processing, and super-resolution imaging systems, surpassing the performance limitations of conventional optical devices^10,11^.

Building on the innovative capabilities of metasurfaces and nanoantennas for precise photon propagation control, Cheng Chi from the Beijing Engineering Research Center of Mixed Reality and Advanced Display and Zhibo Dang from the State Key Lab for Mesoscopic Physics have introduced electron-induced color routers (CRs) in their recent publication in *Light: Science & Applications*^12^. (Fig. 1) These CRs leverage a novel approach by integrating electron beam stimulation with gold nanoantennas to achieve unprecedented control over photon momentum at the nanoscale. Electron-induced CRs combine electron beam stimulation with gold (Au) nanoantennas to control photon momentum at sub-wavelength scales. These nanoantennas with the size of 400 nm × 70 nm serve as platforms for dichromatic photon splitting. By steering the electron impact position within 60 nm, researchers successfully separated green (560 nm wavelength) and red (720 nm wavelength) photon components into distinct angular directions. When the electron beam impacted the edge of the nanoantenna, far-field interference patterns directed photons into different angular regions. In contrast, a central impact produced a non-splitting pattern, illustrating the critical role of impact position in photon manipulation. The modulation mechanism is fundamentally driven by the interference of multipolar moments, such as electric dipole, quadrupole, and toroidal moments, which critically influence the formation of far-field emission patterns. Precise adjustments to the electron beam impact position dynamically modulate these interference effects, thereby facilitating accurate angular splitting of photons. This approach exemplifies the capability to manipulate photon momentum at sub-diffraction scales, unlocking transformative opportunities for advanced photonic applications.

**Fig. 1:**
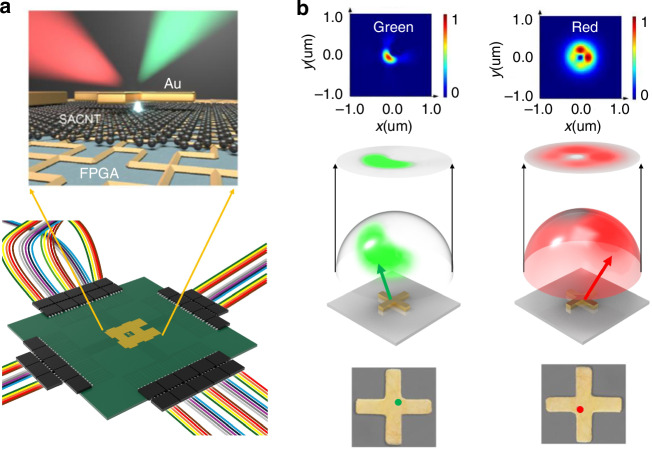
**a** (Top) Schematic of electron-induced color router(CR) and (Bottom) the picture of CRs with field-programmable gate array(FPGA) controller on the PCB board. **b** Symmetrical Au nanoantenna under electron beam stimulation at the nanoantenna corner generates asymmetrical dichromatic dispersion radiation

Electron-induced CRs offer a transformative opportunity for advancing programmable encrypted display devices. (Fig. 2) These displays utilize angular photon splitting patterns to encode information with high resolution and secure data transmission. By leveraging a 4 × 4 programmable matrix and precisely controlling the electron beam impact position within 30 nm, they can switch between “on” and “off” states, enabling robust and reliable data encoding. This reconfigurable functionality supports applications requiring high security, such as encrypted optical systems and advanced communication networks. Moreover, CR arrays demonstrate exceptional potential in on-chip spectroscopy by enhancing both resolution and efficiency in spectral analysis. By exploiting the frequency-dependent angular separation of photons, these arrays enable detailed spectral investigations at sub-wavelength scales, laying the groundwork for compact and integrated spectroscopic platforms. Furthermore, the ability to manipulate photon momentum across multiple frequency channels positions electron-induced CRs as essential components in next-generation quantum information technologies. These systems rely on high-dimensional data encoding and secure communication, both of which are inherently supported by the advanced capabilities of CRs, including the integration of encryption and real-time data retrieval on the optical Fourier plane.

**Fig. 2:**
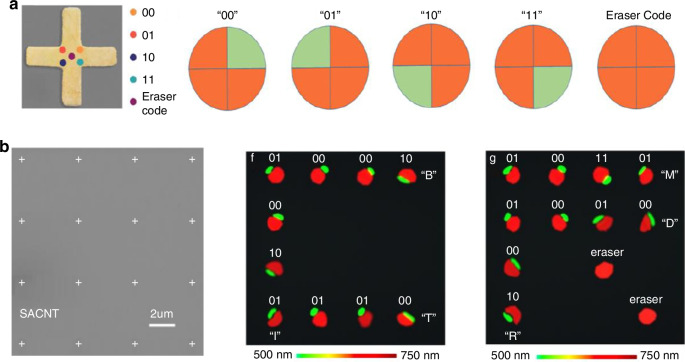
**a** Functional unit for frequency-dependent quaternary encoding. **b** (Left) The scanning electron microscope image of Au cross nanoantenna array. (Center) Two-dimensional encrypted display of capital letters “C”. In the capital letter “C”, the measured splitting patterns of dichromatic components output “01000010”, “01001001” and “01010100” corresponding to ASCII codes of capital letters “B”, “I” and “T”, respectively. (Right) Two-dimensional encrypted display of capital letters “R”. In the capital letter “R”, three character strings of “01001101”, “01010010” and “01000100” are output as capital letters “M”, “R” and “D”, respectively

While electron-induced CRs show potential for practical applications, several challenges need to be addressed to enable their broader use. Material optimization is a primary concern. High plasmonic Q-factor materials such as aluminum (Al) or silver (Ag) could improve performance, particularly at shorter wavelengths, by enhancing optical efficiency and durability. These materials may help expand the applicability of CRs and improve their reliability. Environmental stability is another important challenge. Metallic nanostructures are prone to degradation due to environmental factors such as oxidation, which can reduce functionality over time. Protective coatings or vacuum packaging techniques could be effective strategies to enhance long-term stability and maintain performance. Finally, scalability is a key requirement for further development. Current CR implementations are mainly focused on small-scale devices, and expanding to larger arrays or adapting the technology for integration into more complex systems will be necessary for wider adoption. Addressing these challenges will support the continued advancement of electron-induced CRs in various applications

The advancement of electron-induced CRs represents a pivotal achievement in photonics, enabling precise manipulation of photon momentum at sub-wavelength scales. This innovative capability opens the door to transformative applications, including encrypted displays, on-chip spectroscopy, and quantum information technologies^13–15^. By addressing key challenges such as material optimization, environmental stability, and scalability, this technology holds the potential to reshape the future of photonic and quantum systems. The impact of this research extends well beyond its immediate practical applications. Electron-induced CRs serve as a versatile platform for exploring and controlling photon momentum, laying the groundwork for future interdisciplinary breakthroughs in photonics. Continued research will likely prioritize the development of advanced materials, strategies to ensure long-term stability, and methods to scale CR technology for integration into complex systems. These efforts are expected to drive the emergence of the next generation of optical devices and quantum technologies, fundamentally transforming how we utilize light for information processing, secure communication, and beyond^16^.
